# Supplementary material for: Induction by caterpillars of stored and emitted volatiles in terpene chemotypes from populations of wild cotton (Gossypium hirsutum)
Source: BMC Plant Biol. 2025 Jan 30;25:127. doi: 10.1186/s12870-025-06088-7 (PMC11781055; doi:10.1186/s12870-025-06088-7)
Supplement: Supplementary file 1 — Supplementary Material 1. [file 12870_2025_6088_MOESM1_ESM.pdf]

SUPPLEMENTARY MATERIAL

Induction by caterpillars of stored and emitted volatiles in terpene chemotypes from populations of wild cotton (*Gossypium hirsutum*)

Marine Mamin[1], Mary V Clancy[1], Galien Flückiger[1], Teresa Quijano-Medina[2], Biiniza Pérez-Niño[2], Luis Abdala-Roberts[2], Ted C.J. Turlings[1,3*], Carlos Bustos-Segura[1,4*]

[1] University of Neuchâtel, Institute of Biology, Fundamental and Applied Research in Chemical Ecology, Neuchâtel, Switzerland

[2] Departamento de Ecología Tropical, Campus de Ciencias Biológicas y Agropecuarias, Universidad Autónoma de Yucatán, Mérida, México

[3] State Key Laboratory of Crop Stress Adaptation and Improvement, State Key Laboratory of Cotton Bio-breeding and Integrated Utilization, School of Life Sciences, Henan University, Kaifeng, 475004, China

[4] Present address : Institute of Ecology and Environmental Sciences-Paris, INRAE, Sorbonne Université, CNRS, IRD, Université de Paris, UPEC, Route de St Cyr, 78026 Versailles, France

*Correspondence: ted.turlings@unine.ch; carlos.bustos@inrae.fr

Supplementary figure 1

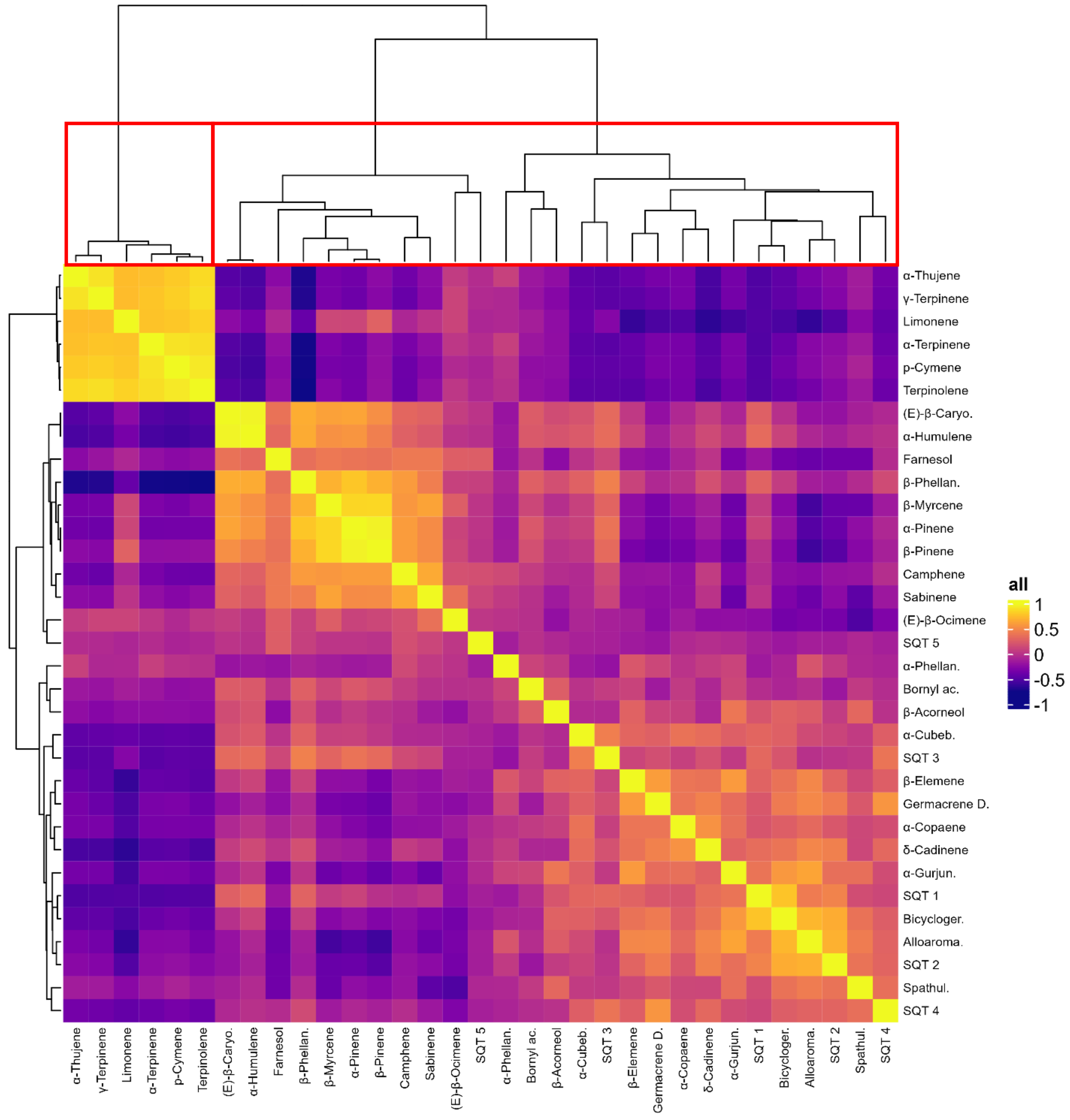

Correlations between relative abundances of stored leaf mono- and sesquiterpenes in undamaged wild cotton plants. Red squares indicate the largest statistically significant clusters (Approximately Unbiased p-values < 0.05).

Supplementary figure 2

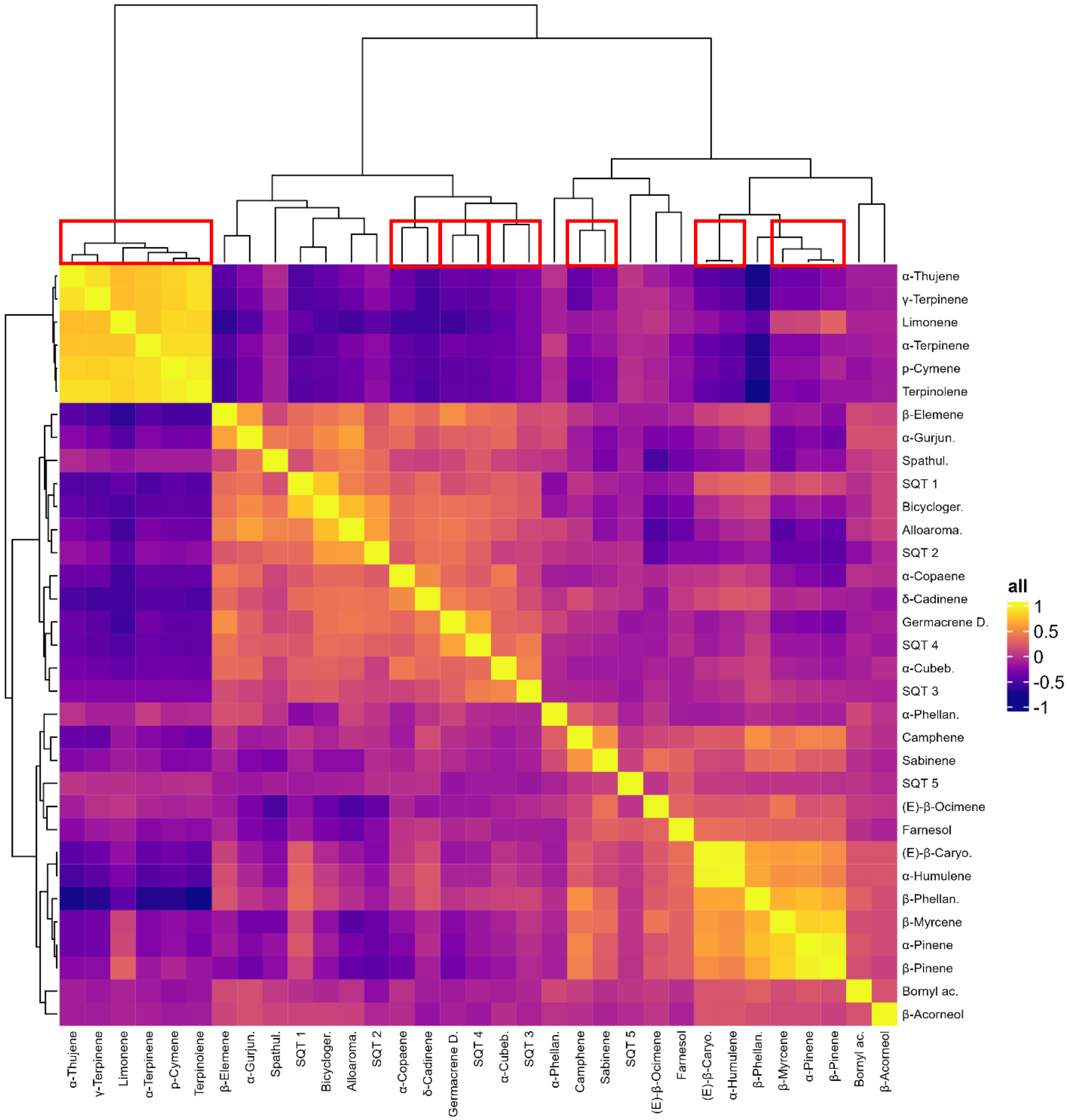

Correlations between relative abundances of stored leaf mono- and sesquiterpenes in wild cotton plants damaged by Spodoptera exigua. Red squares indicate the largest statistically significant clusters (Approximately Unbiased p-values < 0.05)

Supplementary figure 3

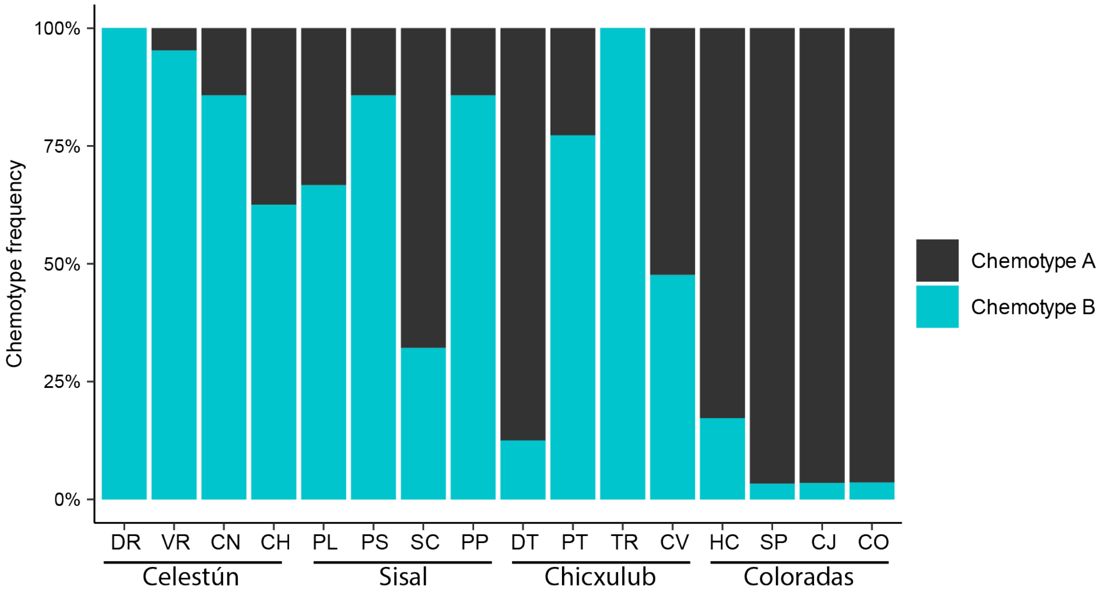

Prevalence of the two chemotypes among the populations.

Supplementary figure 4

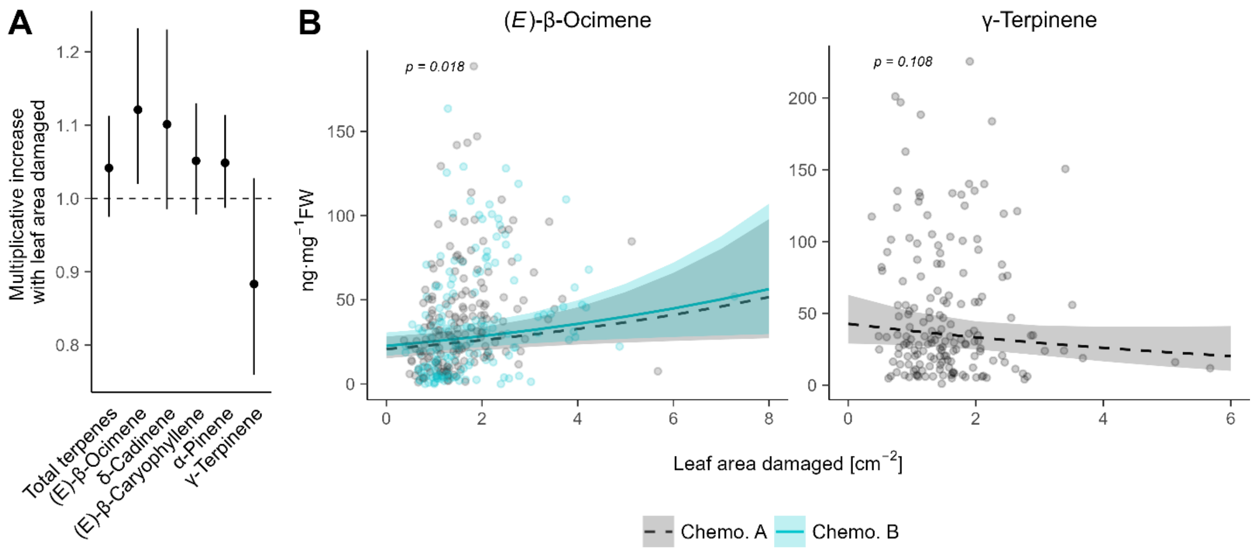

Relationship between damage intensity and accumulation of volatile mono- and sesquiterpenes in leaves of wild cotton. A: multiplicative increases (back-transformed coefficient estimates from GLMMs and their 95% CIs. The dotted line indicates a coefficient of 1, where quantities emitted remain constant with damaged leaf area. B: adjusted predictions and 95% CI from GLMMs with observed values as data points. γ-Terpinene was analysed within chemotype A while all other compounds were analysed for both chemotypes together, with chemotype set as fixed factor.

Supplementary figure 5

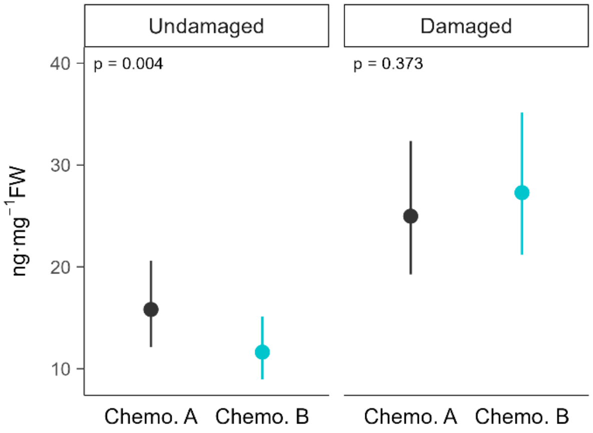

Variation between chemotypes in the leaf concentrations of (*E*)-β-ocimene in undamaged wild cotton and plants damaged by *Spodoptera exigua* for two days. Estimated marginal means and 95% CI from GLMMs performed separately for undamaged and damaged plants. The model on damaged plants includes damage intensity as covariate.

Supplementary figure 6

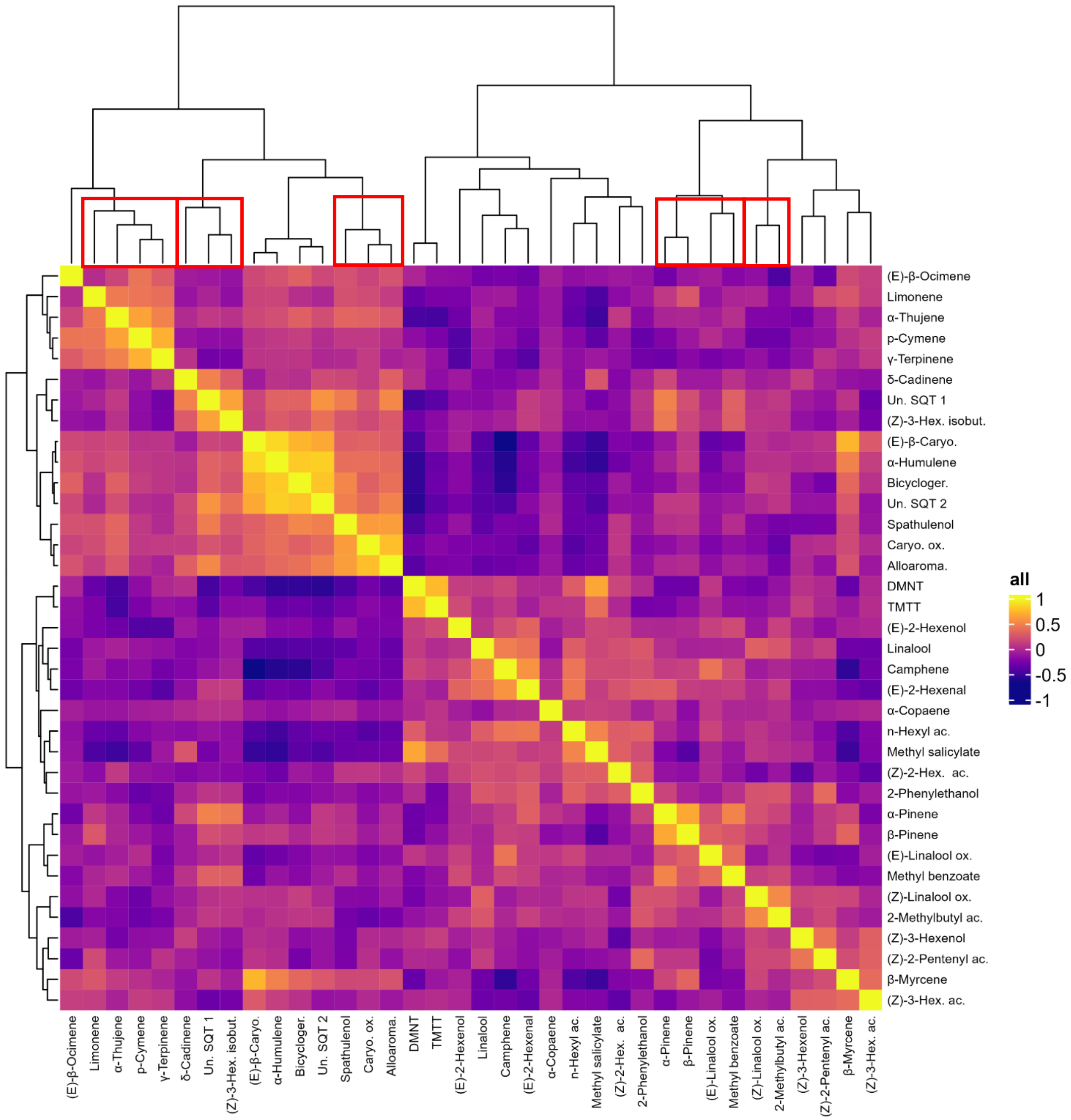

Correlations between relative abundances of all volatiles emitted by undamaged wild cotton plants. Red squares indicate largest statistically significant clusters (Approximately Unbiased p-values < 0.05). Only compounds at least in 10% of the undamaged plants were considered.

Supplementary figure 7

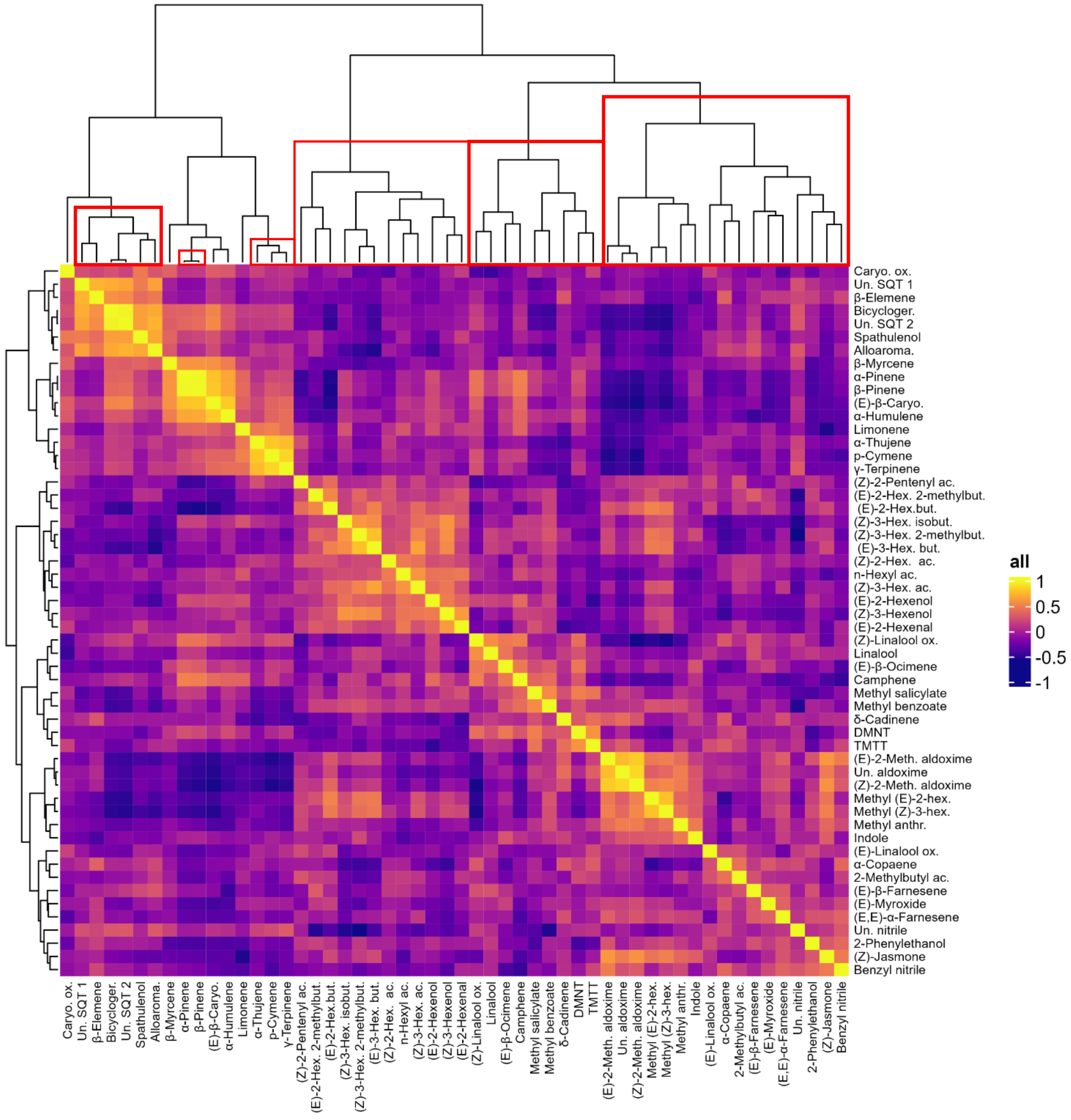

Correlations between relative abundances of all volatiles emitted by wild cotton plants damaged by *Spodoptera exigua*. Red squares indicate largest statistically significant clusters (Approximately Unbiased p-values < 0.05).

Supplementary figure 8

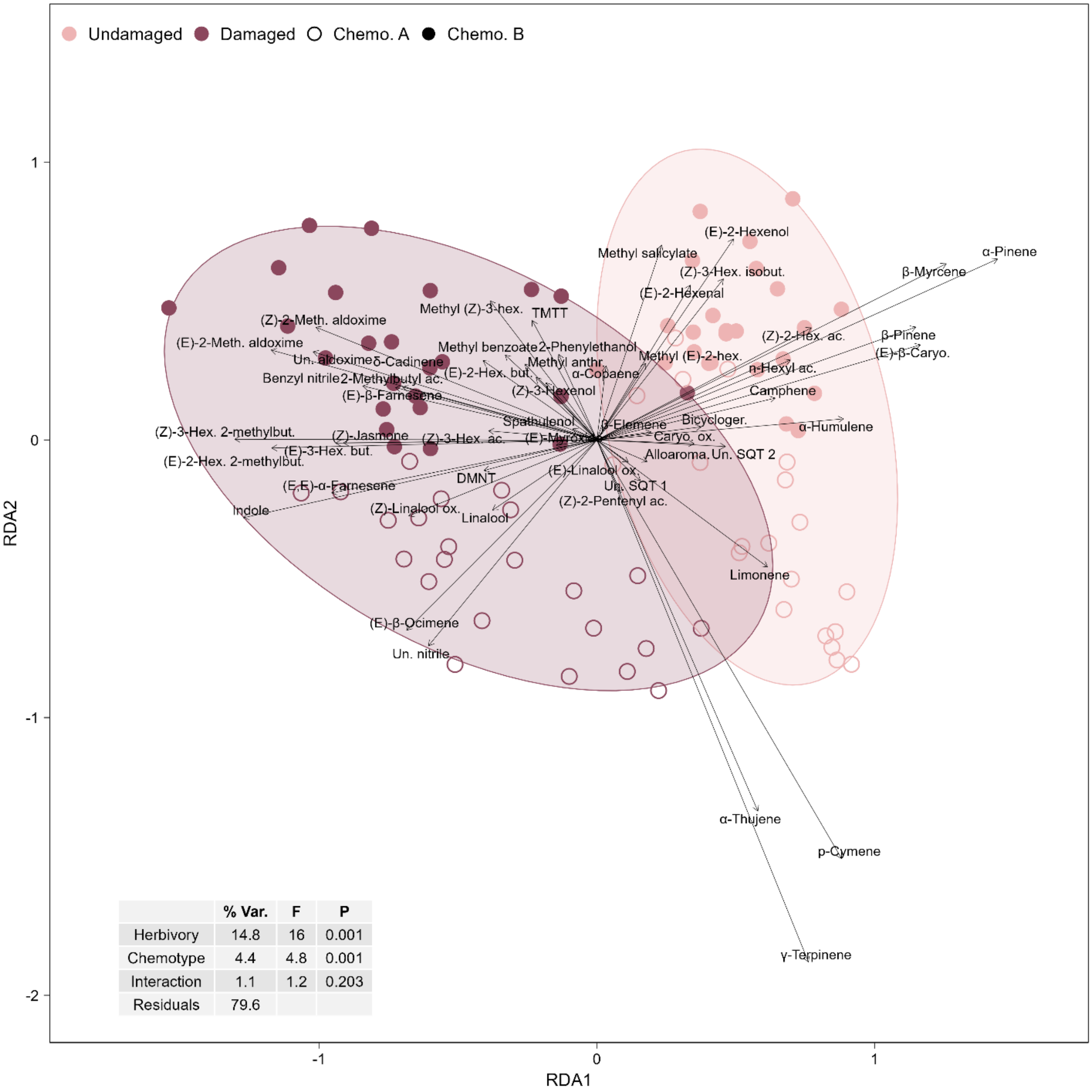

	% Var.	F	P
Herbivory	14.8	16	0.001
Chemotype	4.4	4.8	0.001
Interaction	1.1	1.2	0.203
Residuals	79.6		

Biplot with symmetric scaling of a pRDA on the relative abundances (clr-transformed) of all compounds quantified in the headspace samples. Highly inducible compounds were defined as the ones with a negative score on the first axis.

Supplementary figure 9

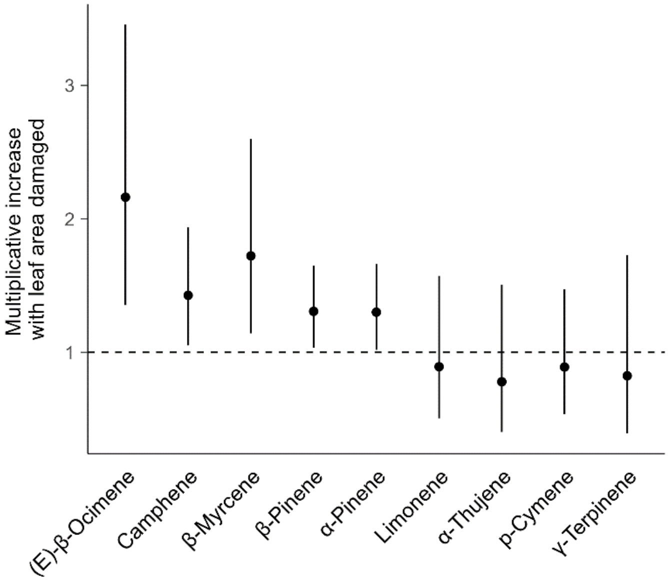

Relationship between damage intensity and emission rates of monoterpenes detected in both headspace and leaf extracts. Multiplicative increases with the leaf area damaged (back-transformed coefficient estimates of GLMMs and their 95% CIs). The dotted line indicates a coefficient of 1, where quantities emitted remain constant with damaged area. γ-Terpinene, p-cymene, α-thujene and limonene were analyzed within chemotype A while all other compounds were analyzed for both chemotypes together, with chemotype set as fixed factor.

Supplementary figure 10

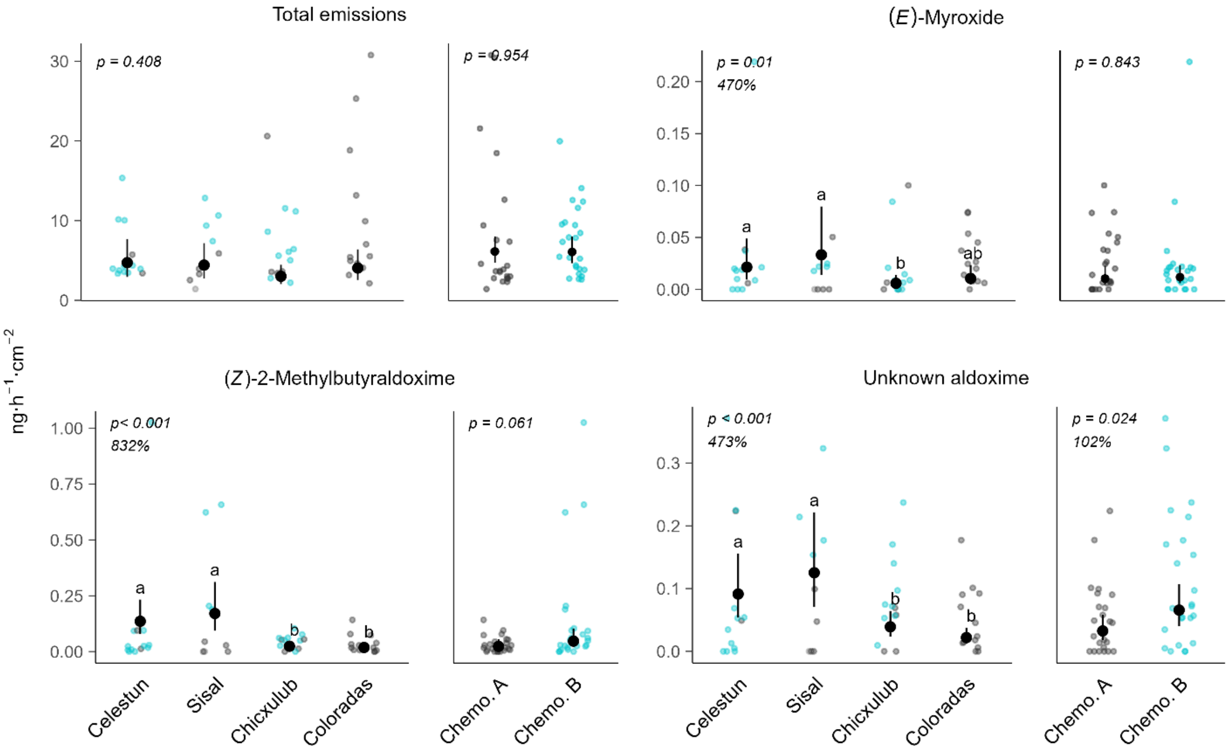

Variation in total emissions of herbivore-induced volatiles (highly induced compounds only) and in emissions of (*E*)-myroxide and aldoximes among the four regions grouping wild cotton populations along the Yucatan coast and between the two leaf-terpene chemotypes. Headspace volatile emissions were sampled from 48 wild cotton plant damaged by *Spodoptera exigua* for two days. Regions are displayed from west to east. Prevalence of chemotypes is nested with regions, as indicated by the color of data points (grey points indicate chemotype A; blue points indicate chemotype B), therefore they were analyzed separately. Estimated marginal means and 95% CI from GLMMs with observed values as data points. Damage intensity was controlled for as covariate. Different letters indicate significant pairwise comparisons after multiple test adjustment (FDR). For regions, % values indicate the difference between the region with the lowest and highest estimated mean emission rates.

Supplementary figure 11

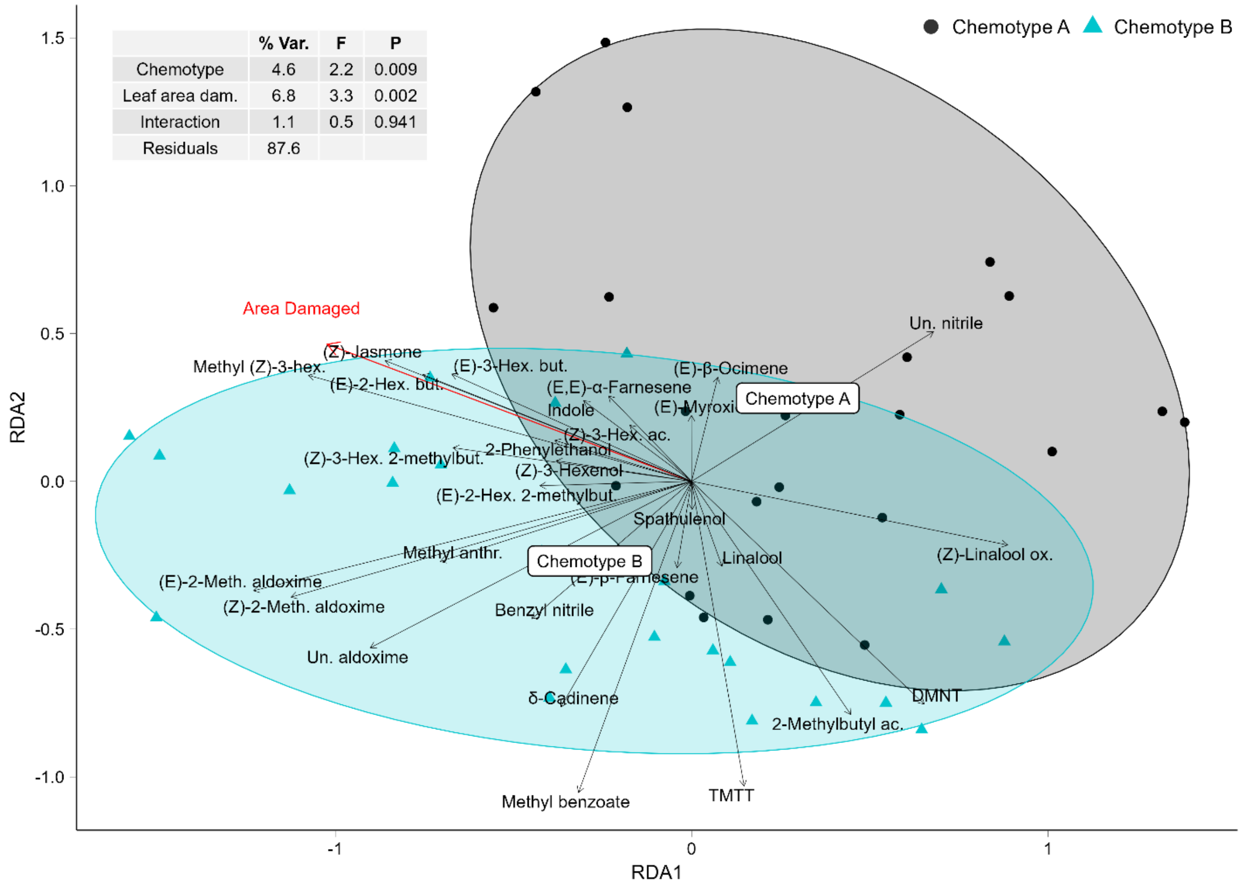

Differences in emission rates of damaged plants between the two chemotypes	
	**Chemotype**
**Unknown nitrile**	$\chi^2_{(1)} = 7.100$, **p = 0.008**
**TMTT**	$\chi^2_{(1)} = 5.971$, **p = 0.014**
Methyl benzoate	$\chi^2_{(1)} = 2.970$, p = 0.085
δ-Cadinene	$\chi^2_{(1)} = 0.450$, p = 0.502
**Benzyl nitrile**	$\chi^2_{(1)} = 4.709$, **p = 0.039**
Methyl anthranilate	$\chi^2_{(1)} = 1.129$, p = 0.288
**Unknown aldoxime**	$\chi^2_{(1)} = 5.069$, **p = 0.024**
**(*Z*)-2-methylbutyraldoxime**	$\chi^2_{(1)} = 3.503$, p = 0.061
**(*E*)-2-methylbutyraldoxime**	$\chi^2_{(1)} = 7.237$, **p = 0.007**
**Inducible compounds differing between chemotypes but detected in undamaged plants: variation in the change with herbivory**	
	**Chemotype x Herbivory**
TMTT	$\chi^2_{(1)} = 1.335$, p = 0.248

Biplot with symmetric scaling of a pRDA on the abundances of highly inducible compounds relative to the whole volatile blend emitted by wild cotton plants damaged by *Spodoptera exigua* (clr-transformed). Highly inducible compounds were defined as described in supplementary figure 7. All compounds are plotted. The table is a summary of the GLMMs analyzing the effect of chemotype on the emission rates of compounds highlighted as potentially varying between chemotypes by the pRDA. Damage intensity was controlled for as covariate in models performed on damaged plants. Chi-squared (χ^2) values are shown, and statistically significant ($p < 0.05$) effects are indicated in bold.

Supplementary figure 12

Biplot with symmetric scaling of a pRDA on the abundances of highly inducible compounds relative to the whole volatile blend emitted by wild cotton plants damaged by *Spodoptera exigua* (clr-transformed). Highly inducible compounds were defined as described in supplementary figure 7. The table is a summary of the GLMMs analyzing the effect of region on the emission rates of compounds highlighted as potentially varying between chemotypes by the pRDA. Damage intensity was controlled for as covariate in models performed on damaged plants. Chi-squared (χ^2) values are shown, and statistically significant ($p < 0.05$) effects are indicated in bold.

**Differences in emission rates of damaged plants among the four regions**	
	**Region**
**Unknown nitrile**	$\chi^2_{(3)}$ = 8.034, **p = 0.045**
(*Z*)-Linalool oxide	$\chi^2_{(3)}$ = 2.400, p = 0.493
DMNT	$\chi^2_{(3)}$ =0 .916, p = 0.821
TMTT	$\chi^2_{(3)}$ = 3.386, p = 0.335
(*E*)-β-Ocimene	$\chi^2_{(3)}$ = 1.284, p = 0.733
Linalool	$\chi^2_{(3)}$ = 3.632, p = 0.304
**δ-Cadinene**	$\chi^2_{(3)}$ = 14.293, **p = 0.002**
Methyl benzoate	$\chi^2_{(3)}$ = 7.670, p = 0.053
**Unknown aldoxime**	$\chi^2_{(3)}$ = 25.789, **p < 0.001**
**(*Z*)-2-methylbutyraldoxime**	$\chi^2_{(3)}$ = 71.290, **p < 0.001**
**(*E*)-2-methylbutyraldoxime**	$\chi^2_{(3)}$ = 62.022, **p < 0.001**
(*E*)-3-Hexenyl butyrate	$\chi^2_{(3)}$ = 3.880, p = 0.274
(Z)-3-Hexenyl 2-methylbutyrate	$\chi^2_{(3)}$ = 3.928, p = 0.269
**(*E,E*)-α-Farnesene**	$\chi^2_{(3)}$ = 25.448, **p < 0.001**
(*E*)-β-Farnesene	$\chi^2_{(3)}$ = 1.823, p = 0.610
**(*E*)-β-Myroxide**	$\chi^2_{(3)}$ = 11.442, **p = 0.009**
Spathulenol	$\chi^2_{(3)}$ = 6.679, p = 0.083
**Inducible compounds differing among regions but detected in undamaged plants: variation in the increase with herbivory**	
	**Region x Herbivory**
δ-Cadinene	$\chi^2_{(3)}$ = 4.809, p = 0.186

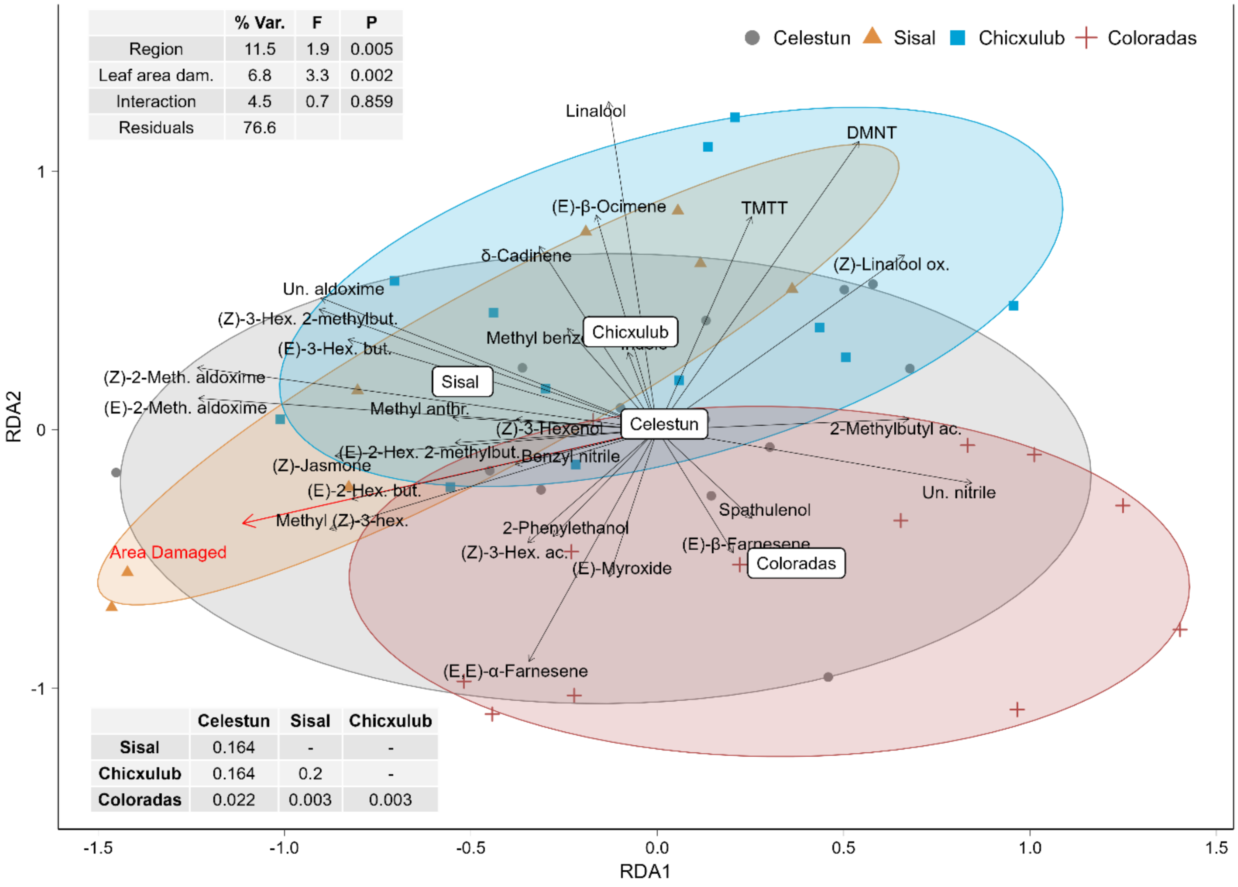

Supplementary table 1

Information about the sites of seeds collection and number of replicates

	Leaf extracts		Headspace			Leaf extracts		Headspace				Leaf extracts	Headspace
**Region**	**Undamaged # Plants**	**Damaged # Plants**	**Undamaged # Plants**	**Damaged # Plants**	**Population**	**Undamaged #Plants**	**Damaged #Plants**	**Undamaged #Plants**	**Damaged #Plants**	**Latitude**	**Longitude**	**# Genotypes/Population**	**# Genotypes/Population**
Celestún	86	79	11	13	Drilos (DR)	20	19	1	4	20.94668	-90.36220	5	3
					Vergel (VR)	21	20	3	3	20.97018	-90.35161	5	3
					Consorcio (CN)	21	20	3	2	20.99828	-90.33714	6	3
					Chapo (CH)	24	20	4	4	21.01394	-90.32414	6	3
Sisal	71	60	9	9	Palapita (PL)	15	9	4	4	21.17525	-89.99657	4	2
					Preseco (PS)	21	19	2	2	21.19097	-89.96278	6	2
					Seco (SC)	28	26	0	1	21.19306	-89.95950	6	1
					Papadzul (PP)	7	6	3	2	21.20333	-89.94119	3	3
Chicxulub	80	70	12	13	Dos Torres (DT)	16	12	3	3	21.29613	-89.57930	6	3
					Pre-Tropical (PT)	22	21	2	3	21.30261	-89.54806	6	3
					Tropical Riviera (TR)	21	18	3	3	21.30396	-89.54164	6	3
					Cinvestav (CV)	21	19	4	4	21.34116	-89.30483	5	4
Coloradas	116	113	13	13	Holchit (HC)	29	28	2	2	21.60842	-88.05024	6	2
					Sin Perros (SP)	30	29	3	3	21.60858	-87.96481	6	3
					Cangrejos (CJ)	29	30	5	5	21.59358	-87.91953	6	3
					Chorlitos (CO)	28	26	3	3	21.58556	-87.89372	6	3
**Sum**	353	322	45	48									

Supplementary table 2

Structures of the GLMMs used to analyze concentrations of volatile terpenes in wild cotton leaves. Chemo = chemotype, Area = fourth leaf area, Damage = damage intensity (total leaf area consumed by *S. exigua*). Full fixed effects are shown, interaction terms were removed if not significantly contributing to the model.

Data	Model	Effects tested with the model	Fixed effects	Random effects (intercepts and slopes)
All plants	A	Herbivory / Chemotype / Chemotype-by-Herbivory interaction	Herbivory + Chemo + Chemo:Herbivory + Area + Batch + Area:Batch	Population + Genotype
	B	Genotype-by-Herbivory interaction	Herbivory + Chemo + Chemo:Herbivory + Area + Batch + Area:Batch	Population + Herbivory:Genotype
	C	Population-by-Herbivory interaction	Herbivory + Chemo + Chemo:Herbivory + Area + Batch + Area:Batch	Herbivory: Population + Genotype
	D	Region-by-Herbivory interaction	Herbivory + Region + Region:Herbivory + Area + Batch + Area:Batch	Population + Genotype
Damaged plants	E	Damage intensity	Damage + Chemo + Chemo:Damage + Area + Batch + Area: Batch	Population + Genotype

Supplementary table 3

Structures of the GLMMs used to analyze emission rates of volatiles by wild cotton plants. Chemo = chemotype, Area = fourth leaf area, Damage = damage intensity (total leaf area consumed by *Spodoptera exigua*). Full models are shown, interaction terms were removed if not significantly contributing to the model.

Data	Model	Effects tested with the model	Structure of the model	
			Fixed effects	Random effects (intercepts)
**All plants**	A	Herbivory / Chemotype-by-Herbivory interaction	Herbivory + Chemo + Chemo:Herbivory + Area + Batch + Area:Batch	Population + Genotype
	B	Region-by-Herbivory interaction	Herbivory + Region + Region:Herbivory + Area + Batch + Area:Batch	Population + Genotype
**Damaged plants**	C	Chemotype / Damage intensity	Chemo + Damage + Chemo:Damage + Area + Batch + Area:Batch	Population
	D	Region	Region + Damage + Region:Damage + Area + Batch + Area:Batch	Population
	E	Population	Chemo + Damage + Chemo:Damage + Area + Batch + Area:Batch	Population

Supplementary table 4

Volatiles quantified in the leaves of undamaged and damaged wild cotton plants (>10% of samples). Median leaf concentrations and quantiles. RI: retention index. RRF: standards used for quantification by relative response factor. Compounds marked with an asterisk were authenticated via comparison to standards.

			Undamaged plants ng mg^{-1} FW					Damaged plants ng mg^{-1} FW				
**Compounds**	**RI**	**RRF**	**Q10**	**Q25**	**Median**	**Q75**	**Q90**	**Q10**	**Q25**	**Median**	**Q75**	**Q90**
**(*Z*)-3-Hexenal**	797	(*Z*)-3-Hexenol	33.189	48.239	64.875	81.373	98.537	71.119	87.934	104.141	123.76	141.217
**(*E*)-2-Hexenal**	849	(*Z*)-3-Hexenol	2.436	3.935	5.524	7.525	8.776	5.407	6.733	8.781	11.033	12.63
**(*E,E*)-2,4-Hexadienal**	910	(*Z*)-3-Hexenol	12.913	22.379	35.004	69.297	96.763	23.89	35.817	49.248	79.42	103.109
**α-Thujene**	929	α-Pinene	0	0.488	0.999	2.241	4.517	0	0	1.087	2.334	6.101
**α-Pinene***	938	α-Pinene	53.242	76.088	113.834	172.234	225.443	57.269	81.222	116.888	178.769	236.72
**Camphene**	955	α-Pinene	0	1.591	2.305	3.324	4.214	0	1.649	2.508	3.607	4.795
**Sabinene**	977	α-Pinene	2.769	4.922	6.892	9.485	13.211	0.53	5.225	7.632	10.132	14.205
**β-Pinene**	983	α-Pinene	10.521	14.325	21.083	32.488	42.822	11.443	16.046	22.252	34.582	47.464
**β-Myrcene***	989	β-Myrcene	43.381	59.826	88.811	133.979	180.069	45.949	62.527	92.878	140.468	180.928
**α-Phellandrene**	1008	α-Pinene	0	0	0.924	1.662	2.633	0	0	1.155	2.188	2.921
**α-Terpinene**	1020	γ-Terpinene	0	0	0	1.459	3.044	0	0	0	1.755	3.924
**p-Cymene**	1028	γ-Terpinene	0	0	0	1.385	3.216	0	0	0	1.787	4.288
**Limonene***	1033	Limonene	3.607	5.357	8.576	16.524	30.263	4.248	6.144	9.929	17.005	39.638
**β-Phellandrene**	1035	γ-Terpinene	0	2.075	4.175	6.455	10.341	0	1.549	4.747	7.168	11.331
**(*E*)-β-Ocimene**	1047	γ-Terpinene	2.62	5.381	13.118	26.512	47.123	4.259	12.729	29.694	51.903	84.456
**γ-Terpinene***	1063	γ-Terpinene	0	0	3.946	32.065	63.088	0	0	5.754	33.838	91.493
**Terpinolene**	1090	γ-Terpinene	0	0	0	2.023	4.06	0	0	0	2.168	5.808
**Bornyl acetate**	1288	γ-Terpinene	0	0	0	0	2.030	0	0	0	0	2.511
**Unknown SQT 1**	1347	(E)-β-Caryophyllene	1.566	2.542	4.899	10.803	15.636	1.892	3.225	5.869	11.153	17.759
**α-Cubebene**	1359	(*E*)-β-Caryophyllene	0	0	0	0	1.246	0	0	0	0	2.02
**α-Copaene**	1389	(*E*)-β-Caryophyllene	0	0	1.477	3.498	5.383	0	0	2.253	4.648	8.343
**β-Elemene**	1401	(*E*)-β-Caryophyllene	0	0	1.245	2.563	3.787	0	0	1.577	3.266	4.511
**α-Gurjunene**	1425	(*E*)-β-Caryophyllene	0	0	0.748	2.19	3.746	0	0	0.82	2.637	4.864
**(*E*)-β-Caryophyllene***	1432	(*E*)-β-Caryophyllene	51.31	90.028	166.794	291.225	387.488	66.058	112.735	186.548	321.953	437.806
**Alloaromadendrene**	1453	(*E*)-β-Caryophyllene	0	0	2.876	7.954	12.19	0	0	3.598	8.396	12.843
**α-Humulene**	1465	(*E*)-β-Caryophyllene	13.338	22.615	44.039	79.025	108.515	16.334	28.611	49.841	90.095	125.651
**Unknown SQT 2**	1473	(*E*)-β-Caryophyllene	0	0.633	1.472	4.444	7.977	0	0.908	1.753	4.28	8.045
**Bicyclogermacrene**	1498	(*E*)-β-Caryophyllene	8.882	17.63	39.568	108.677	165.616	11.89	23.769	49.621	110.859	185.77
**β-Acorneol**	1519	(*E*)-β-Caryophyllene	0	0	0	0.464	0.974	0	0	0	0	0.807
**δ-Cadinene**	1524	(*E*)-β-Caryophyllene	0	1.136	2.401	4.196	6.711	0	1.663	3.051	5.596	8.131
**Germacrene D-4-ol**	1603	(*E*)-β-Caryophyllene	0	0	0	2.209	3.512	0	0	1.171	2.978	4.743
**Spathulenol**	1610	(*E*)-β-Caryophyllene	0	0	0	1.412	3.644	0	0	0	1.671	3.259
**Unknown SQT 3**	1628	(*E*)-β-Caryophyllene	0	0	0	0	2.135	0	0	0	0	2.37
**Unknown SQT 4**	1668	(*E*)-β-Caryophyllene	0	0	0	2.339	4.412	0	0	0	2.971	4.921
**Farnesol**	1749	(*E*)-β-Caryophyllene	0	2.558	4.382	6.489	9.194	0	2.815	4.594	6.954	10.079
**Unknown SQT 5**	2033	(*E*)-β-Caryophyllene	0	2.034	3.253	5.456	8.8	0	2.248	3.533	6.039	9.165

Supplementary table 5

Summary of the GLMMs analyzing the variation in induction (group-by-herbivory interaction) of volatile terpenes concentration in wild cotton leaves among genotypes and populations (random slopes; Likelihood ratio tests on nested models), as well as among geographic regions and between chemotypes (fixed effects; Wald chi-squared tests). Because they were partially nested, the effects of chemotype and region were analysed in separate models. Chemotype was included as fixed effect when testing variation among populations and genotypes. Chi-squared (χ 2) values are shown, and statistically significant ($p < 0.05$) effects are indicated in bold. Df = Degrees of freedom of χ 2 tests. The random slope component includes a term for the variance in slopes and one for the covariance between slopes and intercepts, unless the model could not converge, and the covariance term was excluded (italicized values).

Leaf volatiles	**Genotype x herbivory (χ^2 value, df = 2)**	**Population x herbivory (χ^2 value, df = 2)**	**Region x herbivory (χ^2 value, df = 1)**	**Chemotype x herbivory (χ^2 value, df = 1)**
Total terpenes	0.35	1.22	2.88	0.19
(E)-β-Ocimene	5.23	0.71	2.02	**7.69**
δ-Cadinene	*0.00*	0.88	0.94	0.29
β-Caryophyllene	0.02	0.48	4.00	0.02
α-Pinene	*0.00*	1.42	3.25	0.01
γ-Terpinene	0.04	0.59	1.35	-

Supplementary table 6

Volatiles quantified in the headspace samples collected from undamaged and damaged wild cotton plants (>10% of samples). Median emission rates (normalized by the total leaf area) and quantiles. RI: retention index. RRF: standard used for quantification by relative response factor. Compounds marked with an asterisk were authenticated via comparison to standards. The RI of the unknown aldoxime could not be measured as we had no alkane eluting before that analyte. Distinction between the two 2-methylbutyraldoximes isomers is tentative.

			Undamaged plants ng h^{-1} cm^{-2}					Damaged plants ng h^{-1} cm^{-2}				
**Compounds**	**RI**	**RRF**	**Q10**	**Q25**	**Median**	**Q75**	**Q90**	**Q10**	**Q25**	**Median**	**Q75**	**Q90**
**Unknown aldoxime**	-	(*Z*)-3-Hexenal	0	0	0	0	0	0	0.009	0.054	0.1	0.217
**(*E*)-2-Hexenal***	857	(*E*)-2-Hexenal	0	0	0	0.008	0.014	0	0.012	0.029	0.047	0.093
**(*Z*)-3-Hexenol***	859	(*Z*)-3-Hexenol	0	0	0	0.005	0.034	0.005	0.013	0.02	0.056	0.176
**(*E*)-2-Methylbutyraldoxime***	862	(*E*)-2-Hexenol	0	0	0	0	0	0	0.017	0.081	0.251	0.517
**(*Z*)-2-Methylbutyraldoxime***	868	(*E*)-2-Hexenol	0	0	0	0	0	0	0.007	0.028	0.074	0.156
**(*E*)-2-Hexenol***	871	(*E*)-2-Hexenol	0	0	0	0.004	0.008	0	0.005	0.015	0.029	0.046
**2-Methylbutyl acetate**	881	(*Z*)-3-Hexenyl acetate	0	0	0	0	0.005	0.014	0.023	0.053	0.138	0.315
**(*Z*)-2-Pentenyl acetate**	917	(*Z*)-3-Hexenyl acetate	0	0	0	0.001	0.006	0	0.002	0.011	0.023	0.049
**α-Thujene**	928	α-Pinene	0	0	0	0	0.005	0	0	0.003	0.008	0.021
**α-Pinene***	934	α-Pinene	0.027	0.059	0.117	0.241	0.471	0.183	0.335	0.504	0.863	1.147
**Methyl (*Z*)-3-hexenoate**	936	(*Z*)-3-Hexenyl acetate	0	0	0	0	0	0	0	0.005	0.069	0.263
**Camphene**	949	α-Pinene	0	0	0.005	0.008	0.018	0.006	0.012	0.019	0.033	0.065
**Methyl (*E*)-2-hexenoate**	969	(*Z*)-3-Hexenyl acetate	0	0	0	0	0	0	0	0	0.02	0.05
**β-Pinene***	977	α-Pinene	0	0.007	0.023	0.049	0.096	0.031	0.061	0.095	0.159	0.224
**β-Myrcene***	992	β-Myrcene	0.002	0.016	0.062	0.146	0.279	0.05	0.084	0.165	0.329	0.514
**(*Z*)-3-Hexenyl acetate***	1008	(*Z*)-3-Hexenyl acetate	0	0	0.009	0.029	0.063	0.069	0.11	0.22	0.489	1.023
**n-Hexyl acetate***	1015	(*Z*)-3-Hexenyl acetate	0	0	0	0	0.003	0	0	0.004	0.011	0.02
**(*Z*)-2-Hexenyl acetate**	1018	(*Z*)-3-Hexenyl acetate	0	0	0	0.004	0.006	0	0	0.008	0.013	0.021
**p-Cymene**	1026	α-Pinene	0	0	0	0.009	0.02	0	0	0	0.017	0.029
**Limonene***	1030	α-Pinene	0	0	0.006	0.024	0.052	0	0.012	0.028	0.059	0.104
**(*E*)-β-Ocimene***	1049	(*E*)-β-Ocimene	0	0	0.005	0.023	0.05	0.088	0.141	0.249	0.66	1.279
**γ-Terpinene***	1060	α-Pinene	0	0	0.003	0.033	0.121	0	0.002	0.009	0.117	0.277
**(*Z*)-Linalool oxide**	1074	Linalool	0	0	0	0	0.004	0.032	0.048	0.066	0.106	0.147
**(*E*)-Linalool oxide**	1090	Linalool	0	0	0	0.003	0.007	0	0	0.012	0.03	0.077
**Methyl benzoate***	1097	Benzaldehyde	0	0	0	0	0.005	0.002	0.006	0.014	0.033	0.059
**Linalool***	1100	Linalool	0	0	0	0.005	0.009	0.021	0.032	0.062	0.1	0.143

Supplementary table 6 (cont.)

			Undamaged plants ng h^{-1} cm^{-2}					Damaged plants ng h^{-1} cm^{-2}				
**Compounds**	RI	RFF	**Q10**	**Q25**	**Median**	**Q75**	**Q90**	**Q10**	**Q25**	**Median**	**Q75**	**Q90**
**2-Phenylethanol***	1115	Benzaldehyde	0	0	0	0	0.001	0	0	0.004	0.012	0.023
**DMNT**	1116	Linalool	0	0	0.014	0.043	0.093	0.144	0.2	0.394	0.649	1.049
**Benzyl nitrile**	1140	Benzaldehyde	0	0	0	0	0	0	0.002	0.009	0.022	0.041
**(*E*)-Myroxide**	1142	(*E*)-β-Ocimene	0	0	0	0	0	0	0	0.014	0.025	0.06
**(*Z*)-3-Hexenyl isobutyrate**	1143	(*Z*)-3-Hexenyl acetate	0	0	0	0	0.001	0	0	0.005	0.013	0.036
**Unknown nitrile**	1184	Benzaldehyde	0	0	0	0	0	0	0	0.052	0.122	0.216
**(*E*)-3-Hexenyl butyrate**	1187	(*Z*)-3-Hexenyl acetate	0	0	0	0	0	0.005	0.009	0.023	0.067	0.128
**(*E*)-2-Hexenyl butyrate**	1195	(*Z*)-3-Hexenyl acetate	0	0	0	0	0	0	0	0.006	0.021	0.044
**Methyl salicylate***	1197	Methyl salicylate	0	0	0	0.007	0.014	0	0.002	0.011	0.028	0.076
**(*Z*)-3-Hexenyl 2-methylbutyrate**	1232	(*Z*)-3-Hexenyl acetate	0	0	0	0	0	0.005	0.009	0.03	0.079	0.194
**(*E*)-2-Hexenyl 2-methylbutyrate**	1238	(*Z*)-3-Hexenyl acetate	0	0	0	0	0	0	0.008	0.024	0.051	0.092
**Indole***	1296	Indole	0	0	0	0	0	0.135	0.265	0.576	1.556	3.021
**Unknown SQT 1**	1331	(*E*)-β-Caryophyllene	0	0	0	0	0.002	0	0	0.003	0.008	0.015
**Unknown SQT 2**	1341	(*E*)-β-Caryophyllene	0	0	0.011	0.033	0.044	0.013	0.034	0.057	0.119	0.187
**Methyl anthranilate***	1344	Methyl salicylate	0	0	0	0	0	0	0	0.006	0.014	0.036
**α-Copaene***	1381	(*E*)-β-Caryophyllene	0	0	0	0.003	0.006	0.003	0.011	0.022	0.043	0.09
**β-Elemene**	1396	(*E*)-β-Caryophyllene	0	0	0	0	0	0	0	0	0.008	0.013
**(*Z*)-Jasmone***	1402	(*Z*)-Jasmone	0	0	0	0	0	0	0	0.029	0.085	0.174
**(*E*)-β-Caryophyllene***	1426	(*E*)-β-Caryophyllene	0	0.032	0.142	0.278	0.521	0.145	0.286	0.389	0.605	1.163
**(*E*)-β-Farnesene***	1459	(*E*)-β-Farnesene	0	0	0	0	0	0	0	0.013	0.129	0.28
**α-Humulene***	1460	α-Humulene	0	0.004	0.031	0.076	0.126	0.03	0.059	0.106	0.163	0.314
**Alloaromadendrene**	1467	α-Humulene	0	0	0	0.002	0.009	0	0.004	0.014	0.02	0.038
**Bicyclogermacrene**	1502	α-Humulene	0	0	0.019	0.088	0.138	0.037	0.078	0.17	0.338	0.547
**(*E,E*)-α-Farnesene**	1510	(*E*)-β-Farnesene	0	0	0	0	0	0	0.01	0.055	0.208	0.578
**δ-Cadinene**	1528	α-Humulene	0	0	0	0	0.004	0.002	0.008	0.013	0.028	0.038
**TMTT**	1580	Geranyl acetate	0	0	0.002	0.016	0.082	0.007	0.029	0.084	0.282	0.417
**Spathulenol**	1584	(*E*)-β-Caryophyllene	0	0	0	0.003	0.008	0	0.002	0.009	0.018	0.029
**Caryophyllene oxide**	1590	(*E*)-β-Caryophyllene	0	0	0	0.004	0.016	0	0	0.006	0.011	0.02

Supplementary table 7

Summary of the GLMMs analysing the variation in HIPV emission rates from damaged wild cotton plants among populations (random intercept; Likelihood ratio tests on nested models), as well as among geographic regions and between chemotypes (fixed effects; Wald chi-squared tests). Because they were partially nested, the effects of chemotype and region were analysed in separate models. Chemotype was included as fixed effect when testing variation among populations. Damage intensity was controlled for in all models. Chi-squared (χ 2) values are shown, and statistically significant ($p < 0.05$) effects are indicated in bold. Df = Degrees of freedom. Ce: Celestún, Si: Sisal, Ch: Chixculub, Co: Coloradas; A: chemotype A. B: chemotype B.

HIPV emitted	**Population** (χ^2 **value, df = 1)**	**Region** (χ^2 **value, df = 3)**	**Effect**	**Chemotype** (χ^2 **value, df = 1)**	**Effect**	**Figure**
Total HIPVs	1.34	2.89	-	0.00	-	Fig. S10
(*E*,*E*)-α-Farnesene	**4.23**	**25.45**	Ch < Co ≤ Ce ≤ Si	0.72	-	Fig. 7
(*E*)-Myroxide	**21.88**	**11.44**	Ch ≤ Co ≤ Ce ≤ Si	0.04	-	Fig. S10
(*E*)-Methylbutyraldoxime	**19.25**	**62.02**	Co ≤ Ch < Ce ≤ Si	**7.24**	B > A	Fig. 7
(Z)-Methylbutyraldoxime	**20.86**	**71.29**	Co ≤ Ch < Ce ≤ Si	3.50	-	Fig. S10
Unknown oxime	1.89	**25.79**	Co ≤ Ch < Ce ≤ Si	**5.07**	B > A	Fig. 7
Benzyl nitrile	0.11	**8.83**	Ch ≤ Co ≤ Si ≤ Ce	**4.71**	B > A	Fig. 7
Unknown nitrile	1.55	**8.03**	Ce ≤ Si ≤ Ch ≤ Co	**7.10**	A > B	Fig. 7
